# Impacts of short-term low-level exposure to air pollutants on hospital admissions for pulmonary sepsis in elderly patients

**DOI:** 10.1186/s12890-023-02652-9

**Published:** 2023-11-17

**Authors:** Jing Chen, Aiming Liu, JunJie Dai, Yichen Li, Yu Zhang, Rongchang Chen, Fei Shi

**Affiliations:** 1 Emergency Department, Shenzhen People’s Hospital (The Second Clinical Medical College, Jinan University; The First Affiliated Hospital, Southern University of Science and Technology), Shenzhen, 518020 Guangdong China; 2 Shenzhen National Climate Observatory, Meteorological Bureau of Shenzhen Municipality, Shenzhen, Guangdong China; 3 Key Laboratory of Shenzhen Respiratory Diseases, Institute of Shenzhen Respiratory Diseases, Shenzhen People’s Hospital (The Second Clinical Medical College, Jinan University; The First Affiliated Hospital, Southern University of Science and Technology), Shenzhen, 518020 Guangdong China; 4 Department of Infectious Diseases, Institute of Shenzhen Respiratory Diseases, Shenzhen People’s Hospital (The Second Clinical Medical College, Jinan University; The First Affiliated Hospital, Southern University of Science and Technology), Shenzhen, 518020 Guangdong China

**Keywords:** Low-level exposure, Air pollutants, Pulmonary sepsis, Elderly, Lag-effect

## Abstract

**Background:**

Acute exposures to high levels of air pollutants are thought to be associated with hospitalization of patients with lung infection, while relatively little is known about the association between air pollutants and HOSPITAL ADMISSIONS FOR pulmonary sepsis.

**Objectives:**

To assess the correlation between low-level exposure to air pollutants and the hospitalizations for pulmonary sepsis in elderly patients.

**Methods:**

A total of 249 elderly patients with pulmonary sepsis from January 2018 to December 2020 in Shenzhen people’s hospital were included. The data regarding hospitalizations for pulmonary sepsis, meteorological factors, and daily average levels of air pollutants on single-day lags (Lag0 to Lag7) in Shenzhen were collected. Low-level exposure was defined as the annual means of air pollutants below the levels of the Ambient Air Quality Standard (AAQS) in China (NO. GB3095-2012) and/or Global Air Quality Guidelines (AQG). A time-stratified case-crossover study design approach was used to evaluate the associations between exposure to air pollutants and incidence of the disease, univariate and multivariate logistic regression analysis to analyze the association between levels of air pollutants and hospitalizations for pulmonary sepsis in elderly patients.

**Results:**

Exposure to PM_1_(*P* = 0.007, Lag 2 day; *P* = 0.038, Lag6 day), PM_2.5_(*P* = 0.046, Lag2 day), PM_10_(*P* = 0.048, Lag4 day), and O_3_(*P* = 0.044, Lag6 day) was positively correlated with elevated risk of hospitalizations for pulmonary sepsis. In addition, logistic regression analysis revealed that exposure to PM_1_ (OR = 1.833, 95%CI:1.032 ~ 3.256, Lag6 day) and O_3_ (OR = 2.091, 95%CI:1.019 ~ 4.289, Lag6 day) were the independent risk factors of pulmonary sepsis in elderly patients.

**Conclusion:**

Our results demonstrate that short-term low-level exposure to PM_1_ and O_3_ could elevate the risk of hospitalizations for pulmonary sepsis in elderly patients in Shenzhen, providing evidence for developing early warning and screening systems for pulmonary sepsis.

**Supplementary Information:**

The online version contains supplementary material available at 10.1186/s12890-023-02652-9.

## Introduction

Sepsis [1] is defined as a life-threatening organ dysfunction caused by a dysregulated host response to infection. Sepsis may develop rapidly with severe and dangerous symptoms in older adults, and comes with high morbidity and mortality in the world [2].Infections that lead to sepsis often start in a variety of organs. As the first line of defense against germs, lung is the most common source of infection for sepsis(about > 50%) [3, 4].Although in-patients are often at greater risks in developing pulmonary sepsis, approximately 80% of sepsis cases occur in the community. Thus, there are urgent needs for early recognition and source control to improve outcomes of septic patients presenting to emergency department (ED).

Exposure to atmospheric pollution is one of the major threats to human health. Diseases caused by atmospheric pollution were responsible for an estimated 9 million premature deaths every year [5]. Currently, particulate matter (PM), ozone (O_3_), nitrogen dioxide (NO_2_), sulfur dioxide (SO_2_), and carbon monoxide (CO) are considered to be the five major air pollutants in the atmosphere [6]. Various atmospheric pollutants can induce inflammation and oxidative stress by stimulating the release of inflammatory and pro-inflammatory mediators in the body, and lead to hospitalization of patients with lung infection. In many severe cases, it can unpredictably progress to sepsis or septic shock [7, 8]. Moreover, exposure to higher level of PM_2.5_ and Ozone were associated with increased risk of mortality in patients with sepsis [9, 10]. The air quality index of Shenzhen city has been listed in the top 10 for past seven years in China, but a high incidence of pulmonary sepsis in the elderly was still recorded in Shenzhen in recent years. The lack of data on personal exposure to certain airborne particles may be likely to increase risk of pulmonary sepsis in the elderly.

In this study, we sought to investigate the possible relationship between short-term exposure to airborne particles andpulmonary sepsis in older adults in Shenzhen. This will provide insight into the new clinical strategy for early recognition and management of pulmonary sepsis in elderly patients.

## Methods and information

### Study subjects

We conducted a retrospective study of 249elderly patients withpulmonary sepsisadmitted to the Pulmonary Department, Shenzhen People’s Hospital(the First Affiliated Hospital of Southern University of Science and Technology, the Second Clinical Medical College of Jinan University), from January 2018 to December2020.All subjects enrolled met the following inclusion criteria: (1) study subjects met the 2016 diagnostic criteria for sepsis 3.0 [11]; (2) the primary site of infection in the study subjects was from the lung; 3) ≥ 60 years of age. Exclusion criteria included: (1) Patients with advanced cancer; (2) Patients with irreversible end-stage disease at the time of admission; (3) People who were bedridden or homebound for a long time (4) People who did not live in the Shenzhen within 1 week before the onset of the disease. This research protocol was approved by the Medical Ethics Committee of Shenzhen People’s Hospital, and the requirements for obtaining informed consent were waived.

### Atmospheric pollution and meteorological information

Meteorological data came from the Shenzhen National Climate Observatory.Daily mean temperature (T) and daily mean relative humidity (RH) for Shenzhen during the period January 1,2018to December 30, 2020were collected. The daily mean concentrations of the air pollutants PM_1_, PM_2.5_, PM_10_, SO_2_, NO_2_, O_3_ and CO in Shenzhen from 7 days before to the day of onset of disease were also collected for each patient, and converted into a uniform unit of calculation (µg/m^3^or mg/m^3^).

### Statistical methodology and data analysis

We applied a time-stratified case-crossover study design approach to investigate the associations between exposure and incidence of disease; This approach compares exposure to air pollutants in the same patient at the onset of the disease (onset period) with exposure to air pollutants in the non-onset of the disease (non-onset period). Self-matched case-control studies are effective to control a number of short-term, relatively fixed confounding factors, such as smoking, diet, genetics, living conditions, economics and other individual factors. Moreover, we further control for time-related factors such as time trends, seasonality, and short-term autocorrelation by matching case periods to control periods. This reduces the confounding effect of conditions such as underlying disease and no underlying disease on the results of the data analysis. We applied the time-stratified case-crossover study design approach to investigate the short-term associations between each atmospheric pollutant (including PM_1_, PM_2.5_, PM_10_, NO_2_, SO_2_, CO, O_3_) and the onset of pulmonary sepsis. The pollutant concentration on the day of onset of disease was treated as Lag0, and the pollutant concentration on days 1–7 before onset of disease was treated as Lag 1-lag7. A single pollutant case-crossover study model was established by using Univariate logistic data analysis. The daily mean concentrations of each air pollutant for each year were used as a control to obtain *P* values, odds ratios (OR) and 95% confidence intervals (CI) for the indicators. These results can determine the correlation between single pollutants and the onset of pulmonary sepsis and determine the optimal lag according to the principle of maximum OR. When the results of the single pollutant model analysis were statistically significant, it indicated that there were independent variables that interacted with each other for the air pollutant. These independent variables were fitted to the multi-pollutant model and multivariate logistic regression analysis was performed. This determined whether single pollutants were an independent risk factor for the onset of pulmonary sepsis. A *P* value of less than 0.05 (*P* < 0.05) was considered as a statistically significant difference. The annual average daily concentration of major atmospheric pollutants was presented as mean with interquartile ranges (IQR). In addition, we used t test to compare annual average daily concentration of key air pollutants in Shenzhen with national ambient air quality standards developed by the WHO.

## Results

### Demographic data of all subjects

A total of 249 elderly patients (age ≥ 60 years) diagnosed with pulmonary sepsis were finally included in the study. 63.1% enrolled patients were male, and 36.9% were female. The demographic characteristics of all the patients are include in Table 1.In total, the population mean of ages for all the subjects was 79.1 years. 69 patients (27.7%) had a history of smoking while 180 (72.3%) were non-smokers. The hospital length of stay (LOS) ranged from7 to 20 days. Additionally, we also performed a subgroup analysis of survivors and non-survivors. The average age of survivors was 79.1 ± 8.9 years, compared to 79.2 ± 7.9 years for non-survivors (*P* = 0.932). Also, no significantly differences were found between the 2 subgroups in sex, smoking history and hospital LOS. Of note, there was a significant increase in respiratory complications among non-survivors when compared with survivors (43.5% vs. 26.7%, *P* = 0.013).

**Table 1:** General information of elderly patients with pulmonary sepsis

	Total	survivors	non–survivors	*P*-value
Number of cases, (%)	249	187	62	
Age, years, ($\stackrel{-}{\text{X}}$±SD)	79.1 ± 8.6	79.1 ± 8.9	79.2 ± 7.9	0.932
Male, n (%)	157(63.1)	119(63.6)	38(61.3)	0.740
Female, n (%)	92(36.9)	68(36.4)	24(38.7)	
Smoking history				
Yes, n (%)	69(27.7)	49(26.2)	20(32.2)	0.356
None, n (%)	180(72.3)	138(73.8)	42(67.7)	
Comorbidities				
Diabetes mellitus, n (%)	106(42.6)	78(41.7)	28(45.2)	0.634
Hypertensive disorders, n (%)	145(58.2)	113(60.4)	32(51.6)	0.223
Coronary artery disease, n (%)	60(24.1)	41(21.9)	19(30.6)	0.164
Chronic respiratory disease, n (%)	77(30.9)	50(26.7)	27(43.5)	0.013*
Chronic kidney disease, n (%)	55(22.1)	39(20.9)	16(25.8)	0.415
Chronic liver disease, n (%)	13(5.2)	8(4.3)	5(8.1)	0.320
Cerebrovascular disease, n (%)	80(32.1)	49(26.2)	20(32.3)	0.356
Total length of stay, days, M (Q1, Q3)	12(7,20)	12(8,19)	12(5,22.3)	0.631

* Represent *P* < 0.05, suggest statistical significance

### Study of atmospheric factors at baseline

The annual average daily concentration of major atmospheric pollutants (µg/m^3^) in Shenzhen, during January 2018 to December 2020, were assessed in this study. The 3-year average exposure levels of PM_1_, PM_2.5_ and PM_10_ were 12.07 µg/m^3^, 13.59 µg/m^3^, and 20.38 µg/m^3^, respectively. Compared with WHO Air Quality Guidelines (AQG), the averages of PM_2.5_ and PM_10_ were slightly higher in Shenzhen while the averages of NO_2_, O_3_ and SO_2_ were far below the standard. Compared with Ambient Air Quality Standard (AAQS), only the average of PM_2.5_ were slightly higher in Shenzhen. Furthermore, the annual average daily concentration of each air pollutant was used as a control for further analysis. The descriptive statistics for the air pollutant concentration are presented in Table S1-S2 and Figure S1-S2.

### Correlation analysis of air pollutants and meteorological factors

Considering the potential confounding factors, we performed correlation analysis to assess the relationship between meteorological factors (air humidity, air temperature) and air pollutants, and to estimate the time-lag effects. Due to the long span of time and the large number of air pollutant indicators in this study, the *P* values may be difficult to effectively determine the correlation between the indicators. We used the correlation coefficient (r values) to indicate the direct relationship between the variables based on the previous literatures [12, 13]. The absolute value of r value greater than 0.8was considered as a significant correlation between two variables.

By performing Spearman’s correlation analysis, we found that there were stronger correlations among certain air pollutants including PM_1_, PM_2.5_ and PM_10_(r all > 0.8). Meanwhile, no significant correlations were found between meteorological factors and other air pollutants including NO_2_, SO_2_, CO, and O_3_ (r all < 0.8). More details are shown in Table 2.

**Table 2:** The Spearman’s correlation analysis of atmospheric pollutants and meteorological data

		PM_1_	PM_2.5_	PM_10_	NO_2_	SO_2_	CO	O_3_	Temperature, °C	Relative Humidity, %
PM_1_	r Value	1.000*	0.973*	0.848*	0.676	0.488	0.066	0.227	-0.533	-0.354
	*P* Value	—	0.000	0.000	0.000	0.000	0.003	0.000	0.000	0.000
PM_2.5_	r Value	0.973*	1.000*	0.929*	0.617	0.417	0.069	0.240	-0.443	-0.362
	*P* Value	0.000	—	0.000	0.000	0.000	0.017	0.000	0.000	0.000
PM_10_	r Value	0.848*	0.929*	1.000*	0.492	0.345	0.037	0.216	-0.343	-0.446
	*P* Value	0.000	0.000	—	0.000	0.000	0.199	0.000	0.000	0.000
NO_2_	r Value	0.676	0.617	0.492	1.000*	0.547	0.112	-0.078	-0.444	-0.240
	*P* Value	0.000	0.000	0.000	—	0.000	0.000	0.007	0.000	0.000
SO_2_	r Value	0.488	0.417	0.345	0.547	1.000*	0.044	0.155	-0.360	-0.501
	*P* Value	0.000	0.000	0.000	0.000	—	0.122	0.000	0.000	0.000
CO	r Value	0.066	0.069	0.037	0.112	0.044	1.000*	-0.053	0.003	0.092
	*P* Value	0.003	0.017	0.199	0.000	0.122	—	0.064	0.908	0.001
O_3_	r Value	0.227	0.240	0.216	-0.078	0.155	-0.053	1.000*	0.052	-0.390
	*P* Value	0.000	0.000	0.000	0.007	0.000	0.064	—	0.069	0.000
Temperature, ℃	r Value	-0.533	-0.443	-0.343	-0.444	-0.360	0.003	0.052	1.000*	0.345
	*P* Value	0.000	0.000	0.000	0.000	0.000	0.908	0.069	—	0.000
Relative Humidity, %	r Value	-0.354	-0.362	-0.446	-0.240	-0.501	0.092	-0.390	0.345	1.000*
	*P* Value	0.000	0.000	0.000	0.000	0.000	0.001	0.000	0.000	—

Definition of abbreviations: PM = particulate matter; SO_2_ = Sulfur dioxide; NO_2_ = Nitrogen dioxide; O_3_ = Ozone, CO = Carbon monoxide;

* Represent r > 0.8 or r<-0.8, suggest statistical significance

### Correlation and lag effect of air pollutants with the hospitalization of pulmonary sepsis in elderly patients

The association between pulmonary sepsis and each air pollutant at Lag0-7 were summarized in Tables 3, 4, 5 and 6, which showed the relative risks of hospitalization for pulmonary sepsis was associated with an IQR increase in the air pollutants at different lag days. Over all, exposures to PM_1_(Lag2),PM_1_(Lag6),PM_2.5_(Lag2), PM_10_(Lag4), and O_3_(Lag6)showed strong correlation with the risk of hospitalization in patients with pulmonary sepsis (*P* = 0.007,0.038, 0.046, 0.048, 0.044, respectively).The crude OR (and 95% CIs) at immediate lag day were as follows: PM_1_(Lag2): 2.500 (1.280, 4.883); PM_1_(Lag6): 1.833 (1.032, 3.256); PM_2.5_ (Lag2): 2.222 (1.012, 4.880); PM_10_ (Lag4): 2.429 (1.007, 5.856); O_3_(Lag6): 2.091 (1.019, 4.289).

**Table 3:** Univariate logistics regression analysis of PM_1_ (Lag0-Lag7) and the onset of pulmonary sepsis in elderly patients

	PM_1_		
	OR	95%CI	*P Value*
Lag0	1.227	0.699–2.155	0.485
Lag1	1.350	0.757–2.407	0.314
Lag2	2.500	1.280–4.883	0.007*
Lag3	0.950	0.507–1.780	0.882
Lag4	1.000	0.554–1.806	1.000
Lag5	0.967	0.580–1.610	0.904
Lag6	1.833	1.032–3.256	0.038*
Lag7	1.280	0.759–2.160	0.361

Definition of abbreviations: PM = particulate matter; Lag0 = the day of pulmonary sepsis onset; Lag1 = pulmonary sepsis onset on one day prior; Lag2 = pulmonary sepsis onset on two days prior; Lag3 = pulmonary sepsis onset on three days prior; Lag4 = pulmonary sepsis onset on four day prior; Lag5 = pulmonary sepsis onset on five days prior; Lag6 = pulmonary sepsis onset on six days prior; Lag7 = pulmonary sepsis onset on seven days prior; OR = odds ratio; CI = confidence interval

* represent *P* < 0.05, suggest statistical significance

**Table 4:** Univariate logistics regression analysis of PM_2.5_ (Lag0-Lag7) and the onset of pulmonary sepsis in elderly patients

	PM_2.5_		
	OR	95%CI	*P Value*
Lag0	1.500	0.723–3.114	0.280
Lag1	1.333	0.631–2.818	0.460
Lag2	2.222	1.012–4.880	0.046*
Lag3	1.182	0.529–2.638	0.697
Lag4	1.538	0.765–3.093	0.229
Lag5	1.545	0.724–3.299	0.264
Lag6	1.133	0.566–2.269	0.737
Lag7	1.133	0.566–2.269	0.737

Definition of abbreviations: PM = particulate matter; Lag0 = the day of pulmonary sepsis onset; Lag1 = pulmonary sepsis onset on one day prior; Lag2 = pulmonary sepsis onset on two days prior; Lag3 = pulmonary sepsis onset on three days prior; Lag4 = pulmonary sepsis onset on four day prior; Lag5 = pulmonary sepsis onset on five days prior; Lag6 = pulmonary sepsis onset on six days prior; Lag7 = pulmonary sepsis onset on seven days prior; OR = odds ratio; CI = confidence interval

* Represent *P* < 0.05, suggest statistical significance

**Table 5:** Univariate logistics regression analysis of PM_10_ (Lag0-Lag7) and the onset of pulmonary sepsis in elderly patients

	PM_10_		
	OR	95%CI	*P* Value
Lag0	2.143	0.874–5.256	0.096
Lag1	1.000	0.397–2.519	1.000
Lag2	1.100	0.467–2.590	0.838
Lag3	0.636	0.247–1.642	0.356
Lag4	2.429	1.007–5.856	0.048*
Lag5	1.214	0.599–2.463	0.603
Lag6	1.333	0.631–2.818	0.460
Lag7	1.444	0.617–3.379	0.404

Definition of abbreviations: PM = particulate matter; Lag0 = the day of pulmonary sepsis onset; Lag1 = pulmonary sepsis onset on one day prior; Lag2 = pulmonary sepsis onset on two days prior; Lag3 = pulmonary sepsis onset on three days prior; Lag4 = pulmonary sepsis onset on four day prior; Lag5 = pulmonary sepsis onset on five days prior; Lag6 = pulmonary sepsis onset on six days prior; Lag7 = pulmonary sepsis onset on seven days prior; OR = odds ratio; CI = confidence interval

* Represent *P* < 0.05, suggest statistical significance

**Table 6:** Univariate logistics regression analysis of O_3_ (Lag0-Lag7) and the onset of pulmonary sepsis in elderly patients

	O_3_		
	OR	95%CI	*P Value*
Lag0	1.250	0.648–2.412	0.516
Lag1	1.222	0.656–2.279	0.539
Lag2	1.053	0.562–1.972	0.882
Lag3	1.091	0.612–1.946	0.781
Lag4	0.895	0.465–1.721	0.752
Lag5	1.158	0.627–2.139	0.653
Lag6	2.091	1.019–4.289	0.044*
Lag7	1.105	0.594–2.056	0.765

Definition of abbreviations: O_3_ = Ozone; Lag0 = the day of pulmonary sepsis onset; Lag1 = pulmonary sepsis onset on one day prior; Lag2 = pulmonary sepsis onset on two days prior; Lag3 = pulmonary sepsis onset on three days prior; Lag4 = pulmonary sepsis onset on four day prior; Lag5 = pulmonary sepsis onset on five days prior; Lag6 = pulmonary sepsis onset on six days prior; Lag7 = pulmonary sepsis onset on seven days prior; OR = odds ratio; CI = confidence interval

* Represent *P* < 0.05, suggest statistical significance

To control for potential confounders, we employed multivariate logistic regression in the PM subgroups (Tables 7, 8 and 9). The analysis revealed the levels of PM_1_(Lag2), PM_2.5_(Lag2), and PM_10_(Lag4) correlated strongly to those of other PM, while O_3_ and PM_1_(Lag6) showed no significant correlation in the subgroup. Statistically significant results at immediate lag day were as follows: PM_1_ and PM_10_ at Lag2: 1.067 (95% CI 1.010, 1.127),*P* = 0.021; PM_2.5_ and PM_10_ at Lag2: 1.090 (1.013, 1.174), *P* = 0.021; PM_10_ and PM_1_ at Lag4: 1.073 (1.048, 1.099), *P* = 0.039; PM_10_ and PM_2.5_ at Lag4: 1.059 (1.722, 1.998), *P* = 0.037.Since no correlation was found between O_3_ and potential confounders (shown in Table 3), there was no need to run a multivariate logistic regression model with O_3_ and other variables. Therefore, exposure to PM_1_(Lag6) and O_3_(Lag6) were both independently associated with elevated hospitalization risk in elderly patients with pulmonary sepsis.

**Table 7:** Multivariate logistic regression analysis of PM_1_ (Lag2, Lag6) and the onset of sepsis pulmonary sepsis in elderly patients

	PM_1_(Elderly group)		
	OR	95%CI	*P* Value
Lag2			
PM_10_	1.067	1.010–1.127	0.021*
PM_2.5_	1.104	0.986–1.236	0.087
O_3_	1.005	0.984–1.027	0.610
Lag6			
PM_10_	1.044	0.994–1.096	0.084
PM_2.5_	1.096	0.973–1.234	0.132
O_3_	0.994	0.972–1.015	0.557

Definition of abbreviations: PM = particulate matter; Lag2 = pulmonary sepsis onset on two days prior; Lag6 = pulmonary sepsis onset on six days prior; OR = odds ratio; CI = confidence interval

* Represent *P* < 0.05, suggest statistical significance

**Table 8:** Multivariate logistic regression analysis of PM_2.5_ (Lag2) and the onset of pulmonary sepsis in elderly patients

	PM_2.5_(Elderly group)		
	OR	95%CI	*P* Value
PM_10_	1.090	1.013–1.174	0.021*
PM_1_	0.931	0.845–1.024	0.143
O_3_	1.001	0.983–1.020	0.883

Definition of abbreviations: PM = particulate matter; Lag2 = pulmonary sepsis onset on two days prior; OR = odds ratio; CI = confidence interval

* Represent *P* < 0.05, suggest statistical significance

**Table 9:** Multivariate logistic regression analysis of PM_10_ (Lag4) and the onset of pulmonary sepsis in elderly patients

	PM_10_(Elderly group)		
	OR	95%CI	*P* Value
PM_1_	1.073	1.048–1.099	0.039*
PM_2.5_	1.059	1.722–1.998	0.037*
O_3_	0.993	0.982–1.004	0.210

Definition of abbreviations: PM = particulate matter; Lag4 = pulmonary sepsis onset on four day prior; OR = odds ratio; CI = confidence interval

* Represent *P* < 0.05, suggest statistical significance

## Discussion

Herein, this study found that even short-term exposure to low levels of PM_1_, PM_2.5_, PM_10_ and O_3_was associated with onset and hospitalization of pulmonary sepsis for elderly adults at different lag days, while there was a lack of evidence regarding the impact of exposure to NO_2_, SO_2_, and CO on the incidence of pulmonary sepsis in Shenzhen, China. Moreover, univariate and multivariate analyses identifiedthat individual exposure to PM_1_(Lag6) and O_3_(Lag6) are independent risk factors ofhospitalizationforpulmonary sepsis in elderly patients, which may provide reliable evidences to the prevention and management of pulmonary sepsis. To our knowledge, this study is the first to investigate whether low levels of air pollutants are potential risks of pulmonary sepsis in the elderly.

Previous studies have shown that ambient air pollutants tend to exacerbate symptoms of chronic respiratory diseases such as asthma and chronic obstructive pulmonary disease (COPD) in the elderly population. It was also found that exposure to atmospheric pollution in patients with pneumonia leads to an increased risk of hospital admission, particularly in elderly patients [14]. Meanwhile, long-term exposure to low levels of air pollution was recently found to increase the risks of severe respiratory conditions in the elderly. Therefore, the short-term exposure to atmospheric pollution may have the potential to develop severe pulmonary dysfunction that then lead to sepsis in the aging population. Our study demonstrated that the annual average levels of most air pollutants in Shenzhen, China were below the international standards, so it gave us the opportunity to discuss the relationship between short-term exposure to low level air pollutants and pulmonary sepsis in elderly adults.

As early as in 1996, Harvard six Cities Study showed that PM_2.5_ concentrations were positively associated with non-accidental deaths in the elderly. Our findings are also consistent with the study that exposure to PM_2.5_at Lag2 was significantly correlated with hospitalization of pulmonary sepsis in elderly patients (*P* = 0.046).Meanwhile, exposure levels to PM_1_(Lag2 and Lag6),PM_10_(Lag4),andO_3_(Lag6)were found to be positively associated with pulmonary sepsis in the elderly (*P* = 0.007,0.038, 0.048,0.044, respectively).It was also observed that exposure to PM_1_and O_3_at Lag6 was certified to be an independent risk factor for hospitalization of pulmonary sepsis in elderly patients. A case study in northwest China showed that elderly patients exposed to high concentrations of PM_10_ (91 µg/m^3^ daily average) had an increased risk of acute exacerbations of COPD with an optimal lag of up to 27 days [15]. Tian Y studied 1,183,591 patients with respiratory diseases, and identified that PM_1_ was univariable predictors of increased morbidity of total respiratory diseases (RD) and COPD. Additionally, A first-line flow survey in 184 major Chinese cities revealed that O_3_ caused an increased incidence of pneumonia between 2014 and 2017, and was responsible for a large cost to health systems [16].These authors analyzed the correlation between different lung diseases and long-term exposure to air pollutants, thus making it difficult to compare with our results. In our study, we focused on the short-term delayed impact of air pollutants in relatively low levels on pulmonary sepsis. The pathogenesis may be that PM and O_3_ can stimulate macrophages and epithelial cells in the airway to release various pro-inflammatory mediators (such as IL-1, IL-6 and IL-8), leading to lung inflammation, oxidative stress and lung injury. [17, 18] Our findings suggested that short-term exposure to air pollutants, even at a low level, has a significant association on hospitalization of pulmonary sepsis in elderly patients, thus requiring active prevention and attention.

This study has some limitations. First, it was a single-center study, and the limited sample size and duration of study would be a potential bias which may affect the results of our study. Second, we did not investigate the effect of atmospheric pollutants on pulmonary inflammation in patients with pulmonary sepsis due to limited time, manpower and cost. Third, we did not analyze whether atmospheric pollutants can induce an increased risk of certain bacterial infections. Other factors were not available, such as underlying implications and smoking status. Finally, we did not analyze the impact of different levels of exposure to these pollutants on the severity and prognosis of pulmonary sepsis. Moreover, chronic airway diseases including asthma and chronic obstructive pulmonary disease (COPD) are potential factors contributing to the high incidence of pulmonary sepsis in the elderly in Shenzhen. Therefore, further research should be carried out to reveal the relationship between these complications and air pollution.

In conclusion, short-term exposure to low levels of PM and O_3_plays its role and increase the risk of hospitalizations for pulmonary sepsis in elderly adults. Since ambient air pollution is viewed as a modifiable exposure, effective behavior managements should be adopted to protect the at-risk population. Thus, further longitudinal studies will be required to confirm our results.

### Electronic supplementary material

Below is the link to the electronic supplementary material.

Supplementary Material 1

Supplementary Material 2

Supplementary Material 3

Supplementary Material 4

## Data Availability

The datasets generated and/or analysed during the current study are not publicly available due personal privacy but are available from the corresponding author on reasonable request.
