# Supplementary material for: Impacts of short-term low-level exposure to air pollutants on hospital admissions for pulmonary sepsis in elderly patients
Source: BMC Pulm Med. 2023 Nov 17;23:448. doi: 10.1186/s12890-023-02652-9 (PMC10656823; doi:10.1186/s12890-023-02652-9)
Supplement: Supplementary file 1 — Supplementary Material 1 [file 12890_2023_2652_MOESM1_ESM.docx]

**Table S1. Comparison of the level of air pollutants in Shenzhen with WHO** **Air Quality** **Guidelines (AQG) in 2018-2020**

| **Air pollutants** | **AQG** | **Total Mean** | **IQR** | **2018** | | | **2019** | | | **2020** | | |
| --- | --- | --- | --- | --- | --- | --- | --- | --- | --- | --- | --- | --- |
|  |  |  |  | **Mean** | ***p*** | **M/AQG ratio** | **Mean** | ***p*** | **M/AQG ratio** | **Mean** | ***p*** | **M/AQG ratio** |
| PM_1_(μg/m^3^) | — | 14.31 | 12.07 | 15.45 | — | — | 13.37 | — | — | 14.12 | — | — |
| PM_2.5_(μg/m^3^) | 10 | 18.16 | 13.59 | 18.16 | 0.000* | 1.82 | 16.73 | 0.000* | 1.67 | 19.59 | 0.000* | 1.96 |
| PM_10_(μg/m^3^) | 20 | 26.49 | 20.38 | 24.86 | 0.000* | 1.24 | 21.86 | 0.009* | 1.09 | 32.74 | 0.000* | 1.64 |
| NO_2_ (μg/m^3^) | 40 | 9.19 | 6.71 | 10.31 | 0.000* | 0.26 | 9.90 | 0.000* | 0.25 | 7.41 | 0.000* | 0.19 |
| SO_2_(μg/m^3^) | 20 | 2.74 | 1.10 | 3.25 | 0.000* | 0.16 | 2.55 | 0.008* | 0.13 | 2.44 | 0.000* | 0.12 |
| CO (mg/m^3^) | — | 0.45 | 0.20 | 0.52 | — | — | 0.44 | — | — | 0.38 | — | — |
| O_3_ (mg/m^3^) | 100 | 32.55 | 23.36 | 32.25 | 0.000* | 0.32 | 34.51 | 0.000* | 0.35 | 30.89 | 0.000* | 0.31 |

Definition of abbreviations: PM = particulate matter; SO_2_ = Sulfur dioxide; NO_2_ = Nitrogen dioxide; O_3_ = Ozone, CO = Carbon monoxide; AQG= Global Air Quality Guidelines; IQR= interquartile range.

* Represent *P* < 0.05, suggest statistical significance.
