# Supplementary material for: Impacts of short-term low-level exposure to air pollutants on hospital admissions for pulmonary sepsis in elderly patients
Source: BMC Pulm Med. 2023 Nov 17;23:448. doi: 10.1186/s12890-023-02652-9 (PMC10656823; doi:10.1186/s12890-023-02652-9)
Supplement: Supplementary file 2 — Supplementary Material 2 [file 12890_2023_2652_MOESM2_ESM.docx]

**Table S2. Comparison of the level of air pollutants in Shenzhen with China Ambient Air Quality Standard (AAQS) in 2018-2020**

| **Air pollutants** | **AAQS** | **Total Mean** | **IQR** | **2018** | | | **2019** | | | **2020** | | |
| --- | --- | --- | --- | --- | --- | --- | --- | --- | --- | --- | --- | --- |
|  |  |  |  | **Mean** | ***p*** | **M/ AAQS ratio** | **Mean** | ***p*** | **M/ AAQS ratio** | **Mean** | ***p*** | **M/ AAQS ratio** |
| PM_1_(μg/m^3^) | — | 14.31 | 12.07 | 15.45 | — | — | 13.37 | — | — | 14.12 | — | — |
| PM_2.5_(μg/m^3^) | 15 | 18.16 | 13.59 | 18.16 | 0.000* | 1.21 | 16.73 | 0.001* | 1.12 | 19.59 | 0.000* | 1.31 |
| PM_10_(μg/m^3^) | 40 | 26.49 | 20.38 | 24.86 | 0.000* | 0.62 | 21.86 | 0.000* | 0.55 | 32.74 | 0.000* | 0.82 |
| NO_2_ (μg/m^3^) | 40 | 9.19 | 6.71 | 10.31 | 0.000* | 0.26 | 9.90 | 0.000* | 0.25 | 7.41 | 0.000* | 0.19 |
| SO_2_(μg/m^3^) | 20 | 2.74 | 1.10 | 3.25 | 0.000* | 0.16 | 2.55 | 0.008* | 0.13 | 2.44 | 0.000* | 0.12 |
| CO (mg/m^3^) | — | 0.45 | 0.20 | 0.52 | — | — | 0.44 | — | — | 0.38 | — | — |
| O_3_ (mg/m^3^) | 100 | 32.55 | 23.36 | 32.25 | 0.000* | 0.32 | 34.51 | 0.000* | 0.35 | 30.89 | 0.000* | 0.31 |

Definition of abbreviations: PM = particulate matter; SO_2_ = Sulfur dioxide; NO_2_ = Nitrogen dioxide; O_3_ = Ozone, CO = Carbon monoxide; AAQS = Ambient Air Quality Standard; IQR= interquartile range.

* Represent *P* < 0.05, suggest statistical significance.
