# Supplementary figures and images for: Impacts of short-term low-level exposure to air pollutants on hospital admissions for pulmonary sepsis in elderly patients
Source: BMC Pulm Med. 2023 Nov 17;23:448. doi: 10.1186/s12890-023-02652-9 (PMC10656823; doi:10.1186/s12890-023-02652-9)

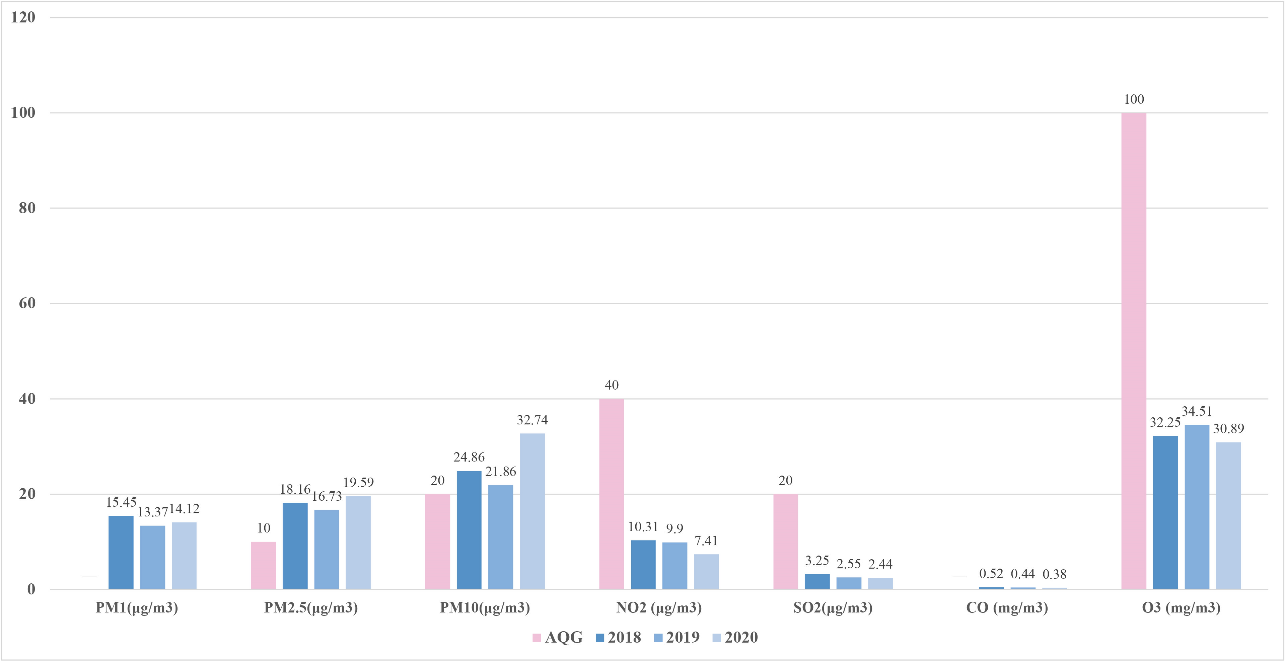


**Figure S1 The level of air pollutants in Shenzhen from 2018 to 2020 and recommended mean AQG level.**

Supplement: Supplementary file 3 — Supplementary Material 3 [file 12890_2023_2652_MOESM3_ESM.docx]

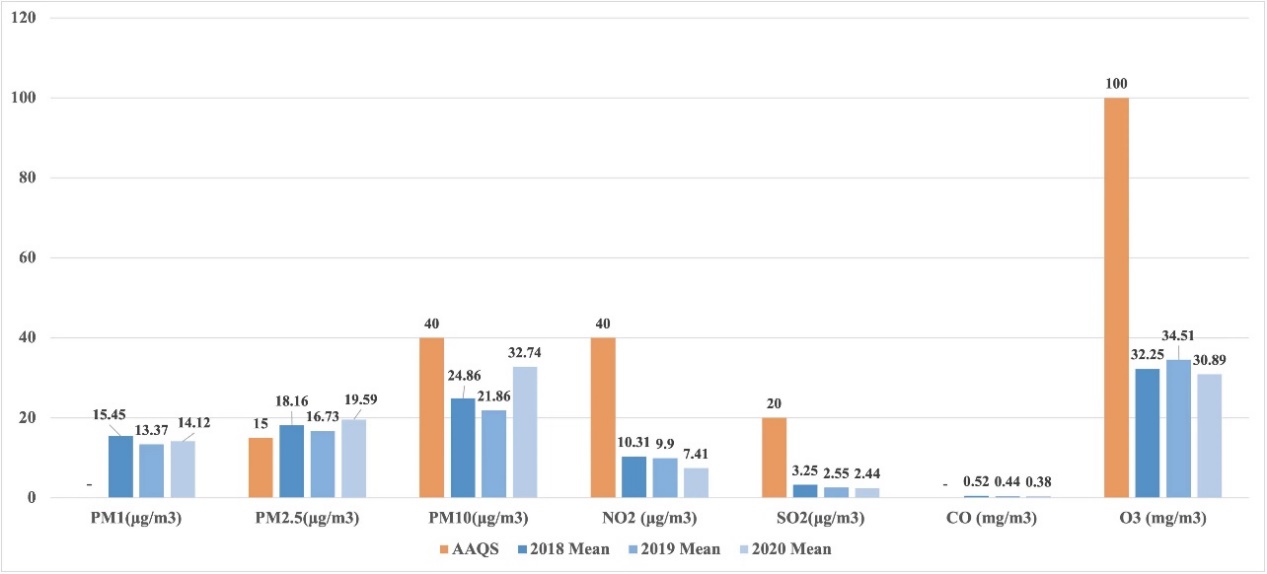


**Figure S2 The level of air pollutants in Shenzhen from 2018 to 2020 and recommended mean AAQS level.**

Supplement: Supplementary file 4 — Supplementary Material 4 [file 12890_2023_2652_MOESM4_ESM.docx]
